# CREM, PRM I and II gene expression in Wistar rats testes treated with antipsychotic drugs: Chlorpromazine, *Rauwolfia vomitoria* and co-administration of reserpine, zinc and ascorbic acid

**DOI:** 10.5935/1518-0557.20200058

**Published:** 2021

**Authors:** Adeleke Opeyemi, Oyewopo Adeoye, Akingbade Adebanji, Johnson Olawumi

**Affiliations:** 1 Department of Anatomy, College of Health Sciences, Osun State University, Osogbo, Osun State, Nigeria); 2 Department of Anatomy, College of Health Sciences, University of Ilorin, Kwara State, Nigeria; 3 Department of Anatomy, College of Medicine and Health Sciences, Ekiti State University, Ekiti State, Nigeria; 4 Department of Anatomy, University of Medical Sciences, Ondo City, Ondo State, Nigeria

**Keywords:** antipsychotics, testes, gene expression, Rauwolfia vomitoria

## Abstract

**Objective:**

The literature has shown that synthetic antipsychotic drugs induce reproductive toxicity, while psychiatric patients treated with traditionally used antipsychotic herbs (*Rauwolfia vomitoria)* showed no traces of reproductive toxicity. Thus, this study aimed to investigate the expression of CREM, PRM I and II genes in the testes of Wistar rats treated with antipsychotic drugs: chlorpromazine, *Rauwolfia vomitoria* (*RV*) and co-administration of reserpine, zinc and ascorbate (RAZ).

**Methods:**

Forty-five adult male Wistar rats with rats with average weight of 180±4.67g were divided into nine groups (A-I) (n=5). Group A was administered saline (control) while rats in Groups B and C received 10 and 20mg/kg body weight (bwt) of chlorpromazine respectively. Groups D and E received 2.5 and 5mg/kg bwt of reserpine, respectively; while Groups F and G received 150 and 300mg/kg bwt of *RV* leaf extract. Groups H and I received (2.5+5+100) mg/kg bwt and (5+10+200) mg/kg of combination of RAZ, respectively for 56 days.

**Results:**

The CREM, PRM I and II genes were significantly downregulated while significant decreased in serum FSH and testosterone concentration were found in the Chlorpromazine- and Reserpine-treated groups. Groups H and I showed a highly significant upregulation of the CREM, PRM I and II genes, and a highly significant increase in serum FSH and testosterone concentrations.

**Conclusion:**

The study concluded that the HPT-Axis was impaired by chlorpromazine and reserpine, while *RV* and a combination of RAZ administration enhanced the axis in an animal model. The study recommended that synthetic antipsychotic drugs should be taken with Zinc and Ascorbate in order to help prevent reproductive toxicity associated with antipsychotic drugs. We need further studies in humans to confirm these findings.

## INTRODUCTION

Infertility is a global health problem, and it is one of the most stressful conditions affecting married couples. Even though not lethal, it has been described as a radical life changing problem that carries with it significant psychological trauma (Oyewopo *et al.,* 2018). Infertility can be caused by various problems in which synthetic antipsychotic drugs are not exempted (Ali & Bhagya, 2011).

Synthetic antipsychotic drugs are chemically synthetized drugs, used in the management of Psychosis. Psychosis is a severe mental disorder, with physical damage to the brain, marked by a deranged personality and a distorted view of reality or an abnormal condition of the mind that involves a loss of contact with reality (Freudenreich, 2012). About 450 million people suffer from mental disorders and one person in four will develop one or more mental or behavioral disorders during their lifetime (WHO, 2003).

Many antipsychotic drugs have been developed over time to combat psychosis. Chlorpromazine (CPZ) is one of the first generation synthetic antipsychotic drugs, and still remains one of the most common drugs used for psychosis treatment worldwide (Chong *et al.,* 2004). Chlorpromazine is a dopamine antagonist of the typical antipsychotic class of medications, possessing additional antiadrenergic, antiserotonergic, anticholinergic and antihistaminergic properties (Healy, 2004). There are many literature reviews reporting that the majority of psychotic patients under Chlorpromazine treatment developed infertility problems, such as diminished libido, erectile dysfunction, impotence, ejaculation inhibition, significant increase in serum prolactin and progesterone, significant decrease in estradiol, testosterone and Luteinizing hormone (Zamani *et al.,* 2015). In an animal model, Raji *et al.* (2005) reported that Chlorpromazine altered the activity of some androgen-dependent enzymes, decreased the weight of rat testes, caput and cauda epididymis and suppressed testicular functions. The Electronic Medicines Compendium (EMC) reported that there is paucity of information as regards to anti-fertility effects of Chlorpromazine in male animals when compared with female animals, where it has been revealed to decrease fertility parameters (EMC, 2019). Ali & Bhagya (2011) reported that Chlorpromazine results in loss of follicle by atresia and delays the onset of puberty in immature female rats.

The search for alternative therapies for the treatment of common diseases has moved man closer to natural products that are easy to assess and affordable. Use of these natural products cannot be overlooked in the treatment of mental and other ailments in Nigeria (Akpanabiatu *et al.,* 2006). *Rauwolfia vomitoria* is one of the natural products used traditionally for the treatment of mental disorders in Nigeria (Akpanabiatu *et al.,* 2006).

*Rauwolfia vomitoria* (*RV*) belongs to the Apocynaceae family. It is mostly found in the forest part of southern Nigeria. The plant is also called swizzle stick in English and is about 8m in height (Orwa *et al*., 2009). The active phytochemical components, as reported by Akpanabiatu *et al.* (2006), are alkaloids, rauwolfine, rescinnamine, serpentine, ajmaline serpentinine, steroid-serposterol, saponin and reserpine - an active compound used in the treatment of psychosis. Similarly, *Rauwolfia vomitoria* leaves extract have been reported to contain high concentrations of Zinc and Vitamin C (Ascorbic acid), elements which are essential to promote male fertility (Ogunlesi *et al.,* 2009). The crude extract of *Rauwolfia vomitoria* has been reported to help in treating swellings in male reproductive organs related to infertility (Sinclair, 2000). It has also been reported to have anti-prostate cancer and anti-diabetic activities, which may serve as fertility enhancers, where infertility is associated with such disorders (Sinclair, 2000).

Nur & Adam (2016) reported that reserpine was one of the first potent antipsychotic drugs isolated from the *Rauwolfia vomitoria* plant. Reserpine mediate depletion of monoamine neurotransmitters in the synapses, which improves antipsychotic behaviors because of its effect, which lasts longer than any other antipsychotic agent (Baumeister *et al.,* 2003). Although, reserpine used only as an antipsychotic drug has been reported to pose some anti-pyramidal side effects (Nur & Adams, 2016). Khazan *et al.* (1960) reported severe atrophy and impaired spermatogenesis in the testes of pigeons treated with reserpine, while Mosad *et al.* (2000) reported mild to severe degenerative changes in rat testis after acute reserpine administration.

The literature has shown that antipsychotic drugs induce reproductive toxicity, but there is dearth of information as regards to the molecular mechanism behind this reproductive toxicity. Moreover, to the best of our knowledge, no study has investigated the effects of the concurrent administration of Reserpine, Ascorbic acid and Zinc (RAZ), the selected phytochemicals present in *Rauwolfia vomitoria* leaves on male reproductive parameters. Thus, this study aimed at assessing the impacts of Chlorpromazine, *Rauwolfia vomitoria* and the co-administration of Reserpine, Ascorbic acid and Zinc (RAZ) on the expression of CREM, PRM I and II genes in the testes of adult Wistar rats.

## MATERIALS AND METHODS

### Compounds procurement and preparation

All compounds used (Chlorpromazine, Reserpine, Zinc and Vitamin C) were pure compounds procured from Hefei TDJ Chemical Co. Ltd China and authenticated at the Pharmacy Department - University of Ilorin, Kwara State. We dissolved 100mg of these compounds in 100ml of distilled water: i.e. 1ml of the solution contains 1mg of the solvent (Stock solution). We let the solution stand for some minutes, constantly shaken for proper dissolution.

### Rauwolfia vomitoria leaves authentication and Ethanolic extraction

*Rauwolfia vomitoria* leaves were locally collected from a farmland in Osogbo and identified in the Division of Botany, Department of Biological Sciences, University of Ilorin, Nigeria. *Rauwolfia vomitoria* leaves were air-dried, the dried pieces were then pulverized using an electric blender (Blender /Miller III, model MS-223, Taiwan, China), and the extraction procedure was done as described by Adeleke *et al.* (2017).

### Experimental Design

Forty-five adult male Wistar rats with an average weight of 180±4.67g were divided into nine groups (A-I) (n=5). Group A was administered saline (control), while rats in Groups B and C received 10 and 20 mg/kg body weight (bwt) of chlorpromazine respectively. Groups D and E received 2.5 and 5 mg/kg bwt of reserpine, respectively; while Groups F and G received 150 and 300 mg/kg bwt of *RV* leaf extract. Groups H and I received (2.5+5+100) mg/kg bwt and (5+10+200) mg/kg of combination of RAZ, respectively for 56 days. Drug administrations were done orally using an orogastric cannula. The rats were fed with pelletized grower mash procured from Mosodun Feeds Nigeria Ltd. Osogbo, with ad libitum access to drinking water. Ethics approval number (UERC/ASN/2017/1067) was gotten from the University of Ilorin ethical review committee (UERC). All procedures were done according to global best practices and institutional guidelines on the care and use of animals.

### Animal Slaughter and Sample Collection

The rats were slaughtered on the 57^th^ day of the experiment, anesthetized with 80mg/kg of ketamine hydrochloride. Blood was withdrawn from the heart apex (left ventricle) for hormonal analysis, while small testicular tissues were excised for Reverse transcriptase Polymerase chain reaction (RT-PCR) analysis.

### Hormone Measuring Assay

Serum levels of Testosterone and Follicle stimulating hormone (FSH) were measured using ELISA kits obtained from Monobind Inc. Lake forest, CA, U.S.A and the procedure was done as described by Adeleke *et al.* (2017).

### Reverse Transcriptase Polymerase Chain Reaction Analysis

RNA was isolated from testis using the TRIzol Reagent (ThermoFisher Scientific). Purified DNA-free RNA was converted to cDNA immediately using ProtoScript® First Strand cDNA Synthesis Kit (NEB). Total cDNA (5µL, 10ng) was subjected to PCR amplification in a 50µL reaction mixture containing 10-µL PCR buffer (10mM Tris-HCl, pH 8.4, 50mM KCl/1.5 mM MgCl2), 2.5µL (10 mM) each, deoxynucleotide triphosphate, 5µL each of forward and reverse (10mM) primers. Amplification conditions were: Pre-denaturation at 94ºC for 5 min, Denaturation at 94ºC for 30 sec, Annealing at 58ºC for 30 sec and Extension at 72ºC for 30 sec then 5 min at 72ºC by 30 cycles. The amplicons generated during the PCR step were resolved on 5% Agarose gel. In-gel expression bands were captured using the iPhone-5c camera (Noir effect). Gel image post-processing was done on the Keynote platform in a MacBook Pro iOS computer. The densitometry analysis was carried out using the Image-J software (2.3.0 V, Mac version), and finally the bar chart showing the relative expression of target genes was done on the Graphpad Prism platform (version 8.03, for Mac iOS).

### Statistical Analysis

We used the GraphPad Prism version 8.03 for all the statistical analyses. All the data was expressed as Mean±SEM while differences among groups were analyzed by one-way ANOVA. Tukey’s test was used to adjust for multiple comparisons. *p*-value <0.05 was considered to be statistically significant.

## RESULTS

### CREM, PROTAMINE I and II gene expression

**Figure 1:**
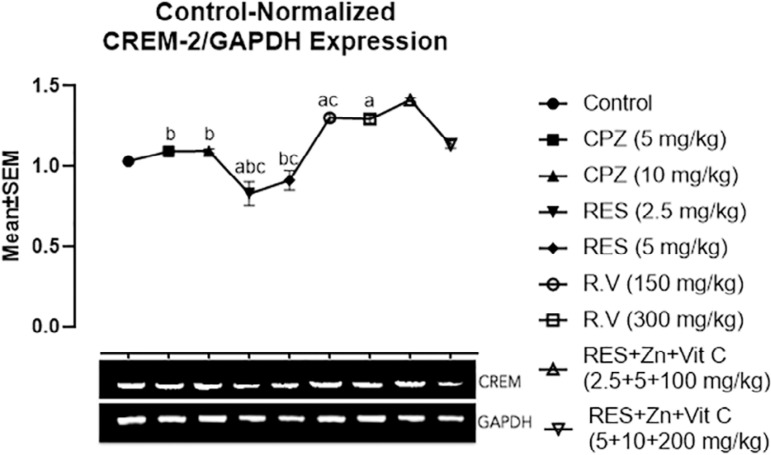
Comparison in CREM gene expression among the groups after the administration of Chlorpromazine, Reserpine, *Rauwolfia vomitoria* and Co-administration of Reserpine, Ascorbate and Zinc. a=Comparison with Control Group A; b=Comparison with Group H and c=Comparison with group I. **p<0.01; ***p<0.001 (n=5).

Results depicted in Figure 1 revealed CREM gene expression mean values after the administration of Chlorpromazine, Reserpine, *Rauwolfia vomitoria* and Co-administration of Reserpine, Ascorbate and Zinc. There was a significantly high upregulation of the CREM gene with the administration of *Rauwolfia vomitoria* and co-administration of Reserpine, Ascorbate and Zinc (Groups F, G and H), when compared with the control group A rats. Moreover, there was a significant downregulation of the CREM gene expression in the 2.5mg/kg reserpine treated group when compared with the control group A rats.

Comparison of the CREM gene expression in the Chlorpromazine, Reserpine and Co-administration of Reserpine, Ascorbate and Zinc treated groups showed high significant down-regulation of CREM gene expression in Chlorpromazine and Reserpine treated groups (Groups B, C, D, and E) when compared with co-administration of 2.5mg/kg Reserpine, 100mg/kg Ascorbate and 5 mg/kg Zinc treated group H. Furthermore, there was a significant downregulation of the CREM in the 2.5 and 5mg/kg-reserpine treated groups D and E, while there was significant up-regulation in the 150mg/kg *Rauwolfia vomitoria* and co-administration of 2.5mg/kg Reserpine, 100mg/kg Ascorbate and 5mg/kg Zinc treated group H when compared with the co-administration of 5mg/kg Reserpine, 200mg/kg Ascorbate and 10 g/kg Zinc treated group I.

**Figure 2:**
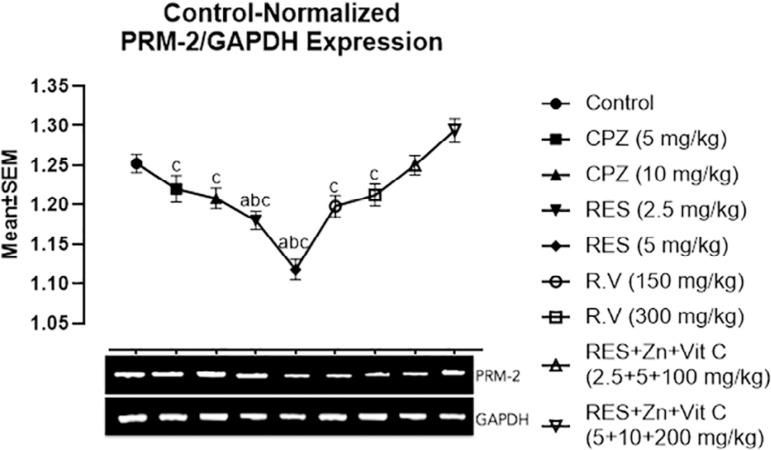
Comparison of the PRM-1 gene expression among the groups after the administration of Chlorpromazine, Reserpine, *Rauwolfia vomitoria* and Co-administration of Reserpine, Ascorbate and Zinc. a=Comparison with Control Group A; b=Comparison with Group H and c=Comparison with group I.*p<0.05; **p<0.01; ***p<0.001 (n=5).

Figure 2 shows the Protamine 1 gene expression mean values among experimental groups, with high significant upregulation of the PRM-1 gene with 10 mg/kg Chlorpromazine, 5 mg/kg Reserpine, 300 mg/kg *Rauwolfia vomitoria* and co-administration of Reserpine, Ascorbate and Zinc treated groups when compared with the control group A rats. In Addition, there was a slightly significant up-regulation in the 150mg/kg *Rauwolfia vomitoria* treated group when compared with the control group A.

There was a highly significant downregulation of the PRM-1 gene expression with 5, 10mg/kg Chlorpromazine, 2.5mg/kg Reserpine and 150mg/kg *Rauwolfia vomitoria* treated groups when compared with the co-administration of 2.5mg/kg Reserpine, 100mg/kg Ascorbate and 5 mg/kg Zinc treated group H. Moreover, a slightly significant downregulation of the PRM-1 gene in the 300mg/kg *Rauwolfia vomitoria* treated groups when compared with co-administration of 2.5mg/kg Reserpine, 100mg/kg Ascorbate and 5mg/kg Zinc treated group H.

Furthermore, Chlorpromazine, Reserpine and *Rauwolfia vomitoria* treated groups B, C, D, E, F and G had a highly significant downregulation of the PRM-1 gene expression when compared with co-administration of 5mg/kg Reserpine, 200mg/kg Ascorbate and 10mg/kg Zinc treated group I.

**Figure 3:**
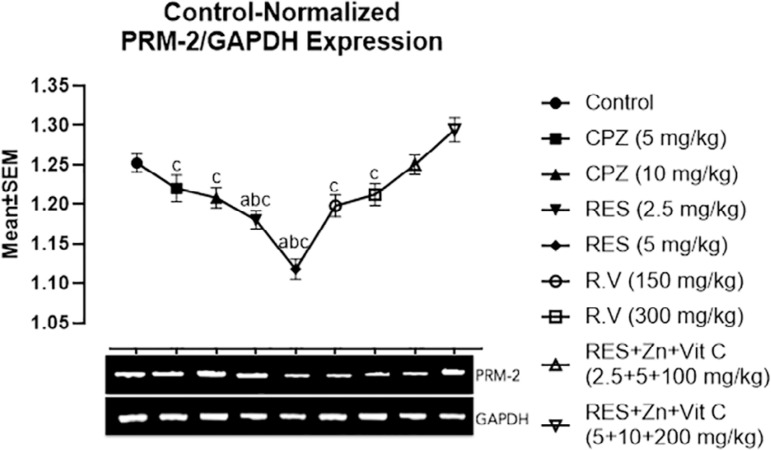
Showed comparison in PRM-2 gene expression among the groups after the administration of Chlorpromazine, Reserpine, *Rauwolfia vomitoria* and Co-administration of Reserpine, Ascorbate and Zinc. a=Comparison with Control Group A; b=Comparison with Group H and c=Comparison with group I.*p<0.05; **p<0.01; ***p<0.001 (n=5).

Figure 3 shows the mean values of Protamine-2 gene expression, revealing a slightly significant downregulation of the PRM-2 gene expression in the 2.5mg/kg Reserpine treated group when compared with the control group A and co-administration of 2.5mg/kg Reserpine, 100mg/kg Ascorbate and 5 mg/kg Zinc treated group H. In addition to this, a highly significant downregulation of the PRM-2 gene expression was found in the 5mg/kg Reserpine treated group when compared with the control group A, and treated group H with co-administration of 2.5mg/kg Reserpine, 100mg/kg Ascorbate and 5mg/kg Zinc.

Comparison of PRM-2 gene expression between the co-administration of 5 mg/kg Reserpine, 200mg/kg Ascorbate and 10mg/kg Zinc treated group I and other treated groups showed significant downregulation of the PRM-2 gene expression in 5, 10mg/kg Chlorpromazine and 300mg/kg *Rauwolfia vomitoria* treated groups. Furthermore, a highly significant down-regulation of the PRM-2 gene expression were seen in the 2.5, 5mg/kg Reserpine and 150mg/kg *Rauwolfia vomitoria* treated groups.

### Hormonal analysis for Follicle Stimulating Hormone and Testosterone

**Figure 4:**
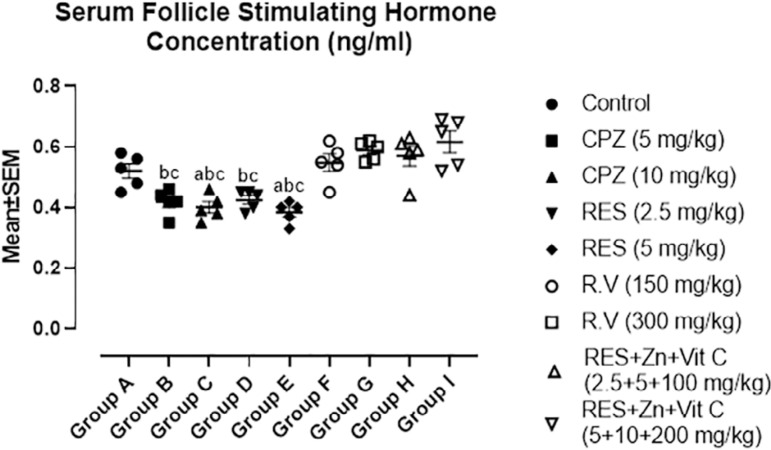
Showed comparison in Serum FSH Concentration among the groups after the administration of Chlorpromazine, Reserpine, *Rauwolfia vomitoria* and Co-administration of Reserpine, Ascorbate and Zinc. a=Comparison with Control Group A; b=Comparison with Group H and c=Comparison with group I.*p<0.05; **p<0.01; ***p<0.001 (n=5).

Result from Figure 4 showed mean values for serum follicle stimulating hormone concentration after administration of Chlorpromazine, Reserpine, *Rauwolfia vomitoria* and Co-administration of Reserpine, Ascorbate and Zinc. Slight significant decrease in serum FSH concentration was observed in 10mg/kg Chlorpromazine treated group while moderate significant decrease was observed in 5mg/kg of Reserpine treated group when compared with the control group A.

Serum FSH concentration was significantly decreased in 5mg/kg and 2.5mg/kg Chlorpromazine and Reserpine respectively when compared with co-administration of 2.5mg/kg Reserpine, 100mg/kg Ascorbate and 5mg/kg Zinc treated group H. Furthermore, high significant decrease in serum FSH were noticed in 10mg/kg Chlorpromazine and 5 mg/kg Reserpine when compared with co-administration of 2.5mg/kg Reserpine, 100mg/kg Ascorbate and 5mg/kg Zinc treated group H. Comparison amongco-administration of 5mg/kg Reserpine, 200mg/kg Ascorbate and 10mg/kg Zinc treated group I with synthetic antipsychotic drugs groups B (5mg/kg Chlorpromazine), C (10mg/kg Chlorpromazine), D (2.5mg/kg Reserpine) and E (5mg/kg Reserpine) showed high significant decrease in their serum FSH concentration when compared with group I.

**Figure 5:**
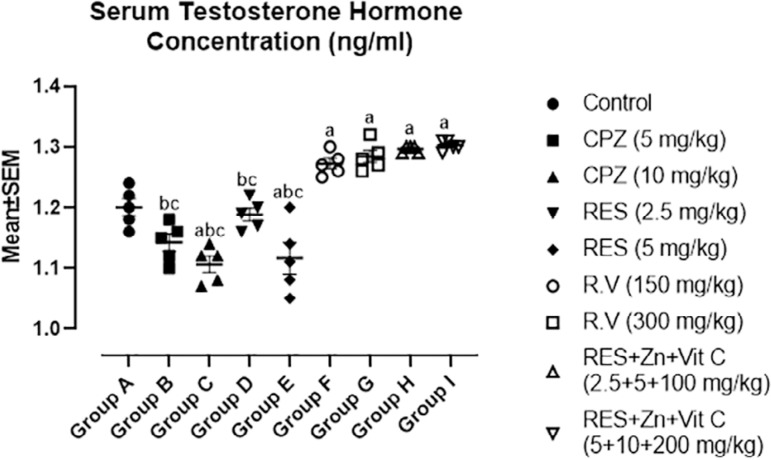
Comparison in Serum Testosterone Concentration among the groups after the administration of Chlorpromazine, Reserpine, *Rauwolfia vomitoria* and Co-administration of Reserpine, Ascorbate and Zinc. a=Comparison with Control Group A; b=Comparison with Group H and c=Comparison with group I.*p<0.05; **p<0.01; ***p<0.001 (n=5).

Figure 5 graph revealed mean values of serum testosterone concentration among the groups after the administration of Chlorpromazine, Reserpine, *Rauwolfia vomitoria* and Co-administration of Reserpine, Ascorbate and Zinc. Serum testosterone were significantly decreased in 10mg/kg Chlorpromazine and 5mg/kg Reserpine treated groups while high significant increase was observed in 300mg/kg *Rauwolfia vomitoria*, co-administration of 2.5mg/kg Reserpine, 100mg/kg Ascorbate and 5mg/kg Zinc and co-administration of 5mg/kg Reserpine, 200mg/kg Ascorbate and 10mg/kg Zinc treated groups when compared with control group A.

Moreover, comparison among co-administration of 2.5mg/kg Reserpine, 100mg/kg Ascorbate and 5mg/kg Zinc treated group A and co-administration of 5 mg/kg Reserpine, 200mg/kg Ascorbate and 10mg/kg Zinc treated group I with synthetic antipsychotic drugs groups B (5mg/kg Chlorpromazine), C (10mg/kg Chlorpromazine), D (2.5mg/kg Reserpine) and E (5mg/kg Reserpine) revealed high significant decrease in serum LH concentration in all synthetic antipsychotic drugs treated groups.

**Figure 6:**
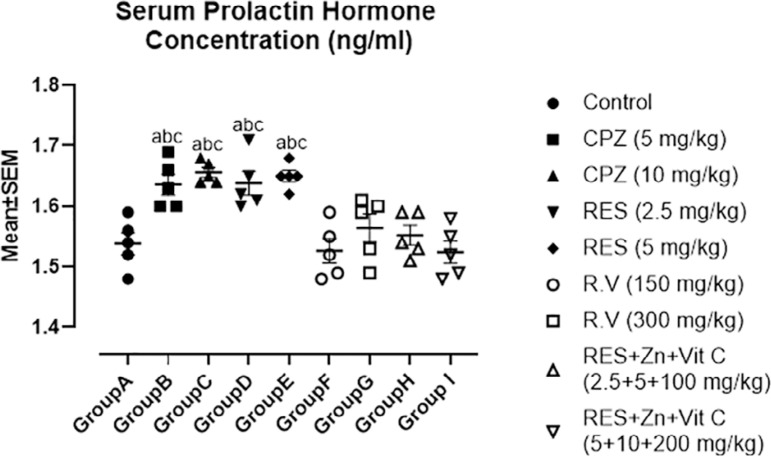
Comparison in Serum Prolactin Concentration among the groups after the administration of Chlorpromazine, Reserpine, *Rauwolfia vomitoria* and Co-administration of Reserpine, Ascorbate and Zinc. a=Comparison with Control Group A; b=Comparison with Group H and c=Comparison with group I.*p<0.05; **p<0.01; ***p<0.001 (n=5).

Prolactin mean values as depicted in Figure 6 revealed high significant increase in serum prolactin concentration in synthetic antipsychotic treated groups B (5mg/kg Chlorpromazine), C (10mg/kg Chlorpromazine), D (2.5mg/kg Reserpine) and E (5mg/kg Reserpine) when compared with the control group A, co-administration of 2.5mg/kg Reserpine, 100mg/kg Ascorbate and 5 mg/kg Zinc and co-administration of 5mg/kg Reserpine, 200mg/kg Ascorbate and 10mg/kg Zinc treated groups. More also, no significant difference was observed among control group A, co-administration of 2.5mg/kg Reserpine, 100mg/kg Ascorbate and 5mg/kg Zinc and co-administration of 5mg/kg Reserpine, 200mg/kg Ascorbate and 10mg/kg Zinc treated groups.

## DISCUSSION

Spermatogenesis has been a complex procedure, involving mitotic, meiosis division and differentiation of spermatogonial stem cells into mature spermatozoa. Phases of spermatogenesis process are mitosis proliferation of spermatogonial stem cells to produce spermatocytes, spermatocyte undergo meiosis division to form haploid round spermatids, while the final stage called spermiogenesis involve conversion of round spermatids to mature elongated spermatids (He *et al.,* 2009). Male infertility (abnormal spermatogenesis) is a pressing issue now, thus promoting an urgent need to solve this fertility deficit. This research study underscores the efficacy of some target antipsychotic compounds in driving the expression of some putative genes involved in normal spermatogenesis.

cAMP responsive element modulator (CREM) is a member of the basic domain-leucine zipper class of transcription factor, which binds as homo and heterodimers to a regulatory palindromic DNA sequence, the cAMP response element (CRE). CRE is localized in the promoter regions of the cAMP responsive genes. The absence of CREM-dependent Transcription in post-meiotic germ cells results in an arrest of spermatid differentiation and apoptosis (Hogeveen & Sassone-Coris, 2006). Several spermatid-specific genes are known to contain a cAMP-responsive element (CRE) serving as a binding site for the transcription factor cAMP-responsive element modulator (CREM) (Sassone-Corsi, 1995). CREM is essential for spermatogenesis, since it has been reported that male mice lacking a functional CREM gene are sterile due to round spermatid maturation arrest (Blendy *et al.,* 1996; Nantel *et al.,* 1996). Infertile men exhibiting round spermatid maturation arrest reveal a substantial reduction or a complete lack at the level of both CREM protein (Weinbauer *et al.,* 1998) and CREM mRNA (Steger, 1999). Due to alternative transcriptional start sites, alternative transcript splicing, and alternative translational start sites, the CREM gene gives rise to functionally different proteins with either activating or repressing potential on target gene expression (Daniel *et al.,* 2000; Behr & Weinbauer, 2001; Gellersen *et al.,* 2002). Weinbauer *et al.* (1998) reported that the alterations in CREM expression, which interfere with the spermatid maturation within a number of cases in idiopathic male infertility.

Protamines are post-meiotic nuclear proteins, which are rich in Arginine. At late haploid phase of spermatogenesis, protamine substitute histones and help in stabilizing sperm DNA and sperm head condensation. During spermatogenesis, haploid spermatids will experience a transformation in its chromatin composition and compactness (Steger, 1999), whereas the deoxynucleic acid (DNA)-histone bond in the round spermatid will be substituted with transition proteins; while the transition protein in elongated spermatids will be substituted with protamine. Thus, changing from histone to protamine stimulates spermatozoa chromatin condensation (Steger *et al.,* 2001). Protamine has been experimentally documented to be essential in male's fertility. Insufficient PRM-1 and PRM-2 concentrations have been implicated in subfertile or severe infertile condition (Oliva, 2006).

From this section we investigate the gene expression pattern of CREM, PRM I and II among the groups after the administration of Chlorpromazine, Reserpine, *Rauwolfia vomitoria* and Co-administration of Reserpine, Zinc and Ascorbate. There was significant downregulation of the CREM and PRM II gene expression in the Chlorpromazine and Reserpine treated groups when compared with the control group. This result is in line with studies from Raji *et al*. (2005) and Khazan *et al*. (1960), who reported anti-fertility effects of Chlorpromazine and Reserpine respectively. Moreover, there was a slightly significant upregulation of the CREM, PRM I and II genes in groups F and G treated with traditionally used antipsychotic drugs (*Rauwolfia vomitoria* leaves extract). Furthermore, groups H and I treated with the co-administration of Reserpine, Zinc and Ascorbate showed highly significant up-regulation of the CREM, PRM I and II genes expression. The CREM gene expression upregulation in groups F, G, H and I might be the result of antioxidant compounds (Zinc and Ascorbate) being part of the drugs constituent administered to these groups. This result is in agreement with those from Ahmadi *et al.* (2016) and Shabanian *et al.* (2017), who reported ascorbic acid to be an important antioxidant that helps prevent sperm defects and boosts sperm motility. Likewise, Ahmadi *et al.* (2016) and Fallah *et al.* (2018) reported efficacy of Zinc supplements in improving sperm count, motility, form, function, quality and fertilizing capacity.

There are reports that CREM alteration or deficiency affect protamine expression; thereby leading to infertility because of disorder in the round spermatid maturation (Blendy *et al.,* 1996). The results from the gene-regulatory based approach in the current study correlate with the reported connection with the putative genes that enhance male normal fertility by Blendy *et al.* (1996).

Serum FSH and Testosterone concentrations were significantly decreased in the high dose synthetic antipsychotic treated groups C and E (Chlorpromazine and Reserpine respectively); while there was no significant difference in the *Rauwolfia vomitoria* treated groups F and G when compared with the control group A. Moreover, there was a highly significant increase in Serum FSH and Testosterone concentrations with the co-administration of Reserpine, Zinc and Ascorbate. FSH and testosterone concentrations found in these groups were in accordance with the reports from Zamani *et al.* (2015), who reported a significant increase in serum prolactin and a significant decrease in testosterone and luteinizing Hormone after Chlorpromazine treatment in rats. Furthermore, Serum Prolactin concentrations in high dose Chlorpromazine and Reserpine treated groups (C and E) were highly significantly increased, and this report is in line with Ben-Jonathan & Hnasko (2001), who reported that dopamine depletion reaching the lactotroph cells resulted in hyperprolactinemia, which always has negative feedback on hypothalamic GnRH secretion.

Reproductive toxicity induced by Chlorpromazine and Reserpine from the results above might come from dopaminergic fibers projecting from A13 and A14 of the ventral tegmental area to the Paraventricular and medial preoptic nuclei of the hypothalamus, respectively (Weiner & Molinoff, 1989). Thus, inhibition or depletion in dopamine concentrations in these hypothalamic areas, because of antipsychotic drugs, may alter pulsatile production of GnRH in the media preoptic area, with its resultant effects felt on the FSH and LH production in the adenohypophysis. FSH has been reported to be a key player in the transcription process of the CREM gene, absence of CREM-dependent transcription in post-meiotic germ cells results in an arrest of spermatid differentiation and apoptosis (Sassone-Corsi, 1998). FSH predominantly regulates the CREM mRNA level. FSH binds with the G-protein alpha-s-coupled receptors, such as Follicle stimulating hormone receptor (FSHR) and activates Adenylate cyclase, Protein kinase A and cAMP-dependent PKA.

The CREM gene consists of CRE regions in the promoters, and its expression is regulated by another cAMP-responsive element binding to protein 1 (CREB1); CREM expression is alternatively regulated by an autoregulation pathway (Monaco *et al.,* 1995; Hogeveen & Sassone-Coris, 2006). A germ cell-specific transcriptional co-activator with four and a half LIM domains, with 5 (ACT) interactions, controls CREM activity. ACT capacity to control CREM activity is regulated by a germ cell-specific kinesin, the Kinesin family member 17 (KIF17), which regulates the ACT subcellular localization. KIF17 colocalizes with ACT in haploid spermatids and mediates the ACT transport from the nucleus to the cytoplasm at specific stages of the spermatid maturation. KIF17 movement is modulated by PKA phosphorylation (c-AMP-dependent). The ability of KIF17 to shuttle between the nuclear and the cytoplasmic compartments and to transport ACT are dependent on neither its motor domain nor on microtubules (Kotaja *et al.,* 2005; Hogeveen & Sassone-Coris, 2006). Thus, CREM activation by ACT is responsible for the transcription of many key genes in postmeiotic germ cells, such as PRM I and PRM II, which are both responsible for DNA condensation and spermatid quality during spermatogenesis.

## CONCLUSION

This present study has elucidated the reproductive toxicity induced by synthetic antipsychotic drugs (Chlorpromazine and Reserpine) and it also gives credence to the activity of compounds present in the traditionally used antipsychotic herb (*Rauwolfia vomitoria*) via crude extract administered, and the concurrent administration of isolated phytochemicals (Reserpine, Zinc and Ascorbate) to improve the menace of infertility associated with antipsychotic drugs by upregulating the CREM, PRM I and II signaling pathways in an animal model. Thus, we concluded that healthy and quality spermatozoa are essential for fertilization. Thus, alteration in the signaling pathways of the genes responsible for spermatogenesis may result in the production of low quality spermatozoa.

## References

[r1] Adeleke OS, Falana BA, Babawale GS, Atere TG, Abayomi TA, Tokunbo OS (2017). Evaluation of the comparative effects of antihypertensive drugs: Methyldopa and Moringa oleifera leaves on the hypothalamic-pituitary-gonadal axis in male wistar rat. J Exp Clin Anat..

[r2] Ahmadi S, Bashiri R, Ghadiri-Anari A, Nadjarzadeh A (2016). Antioxidant supplements and semen parameters: An evidence based review. Int J Reprod Biomed (Yazd).

[r3] Akpanabiatu MI, Umoh IB, Eyong EU (2006). Influence of Rauwolfia vomitoria root bark on cardiac enzymes of normal Wistar albino rats. Recent Prog Med Plants..

[r4] Ali AMH, Bhagya M (2011). Effects of chlorpromazine on the onset of puberty and follicular development in immature female rats. Toxicol Environ Health Sci..

[r5] Baumeister AA, Hawkins MF, Uzelac SM (2003). The myth of reserpine-induced depression: role in the historical development of the monoamine hypothesis. J Hist Neurosci..

[r6] Behr R, Weinbauer GF (2001). cAMP response element modulator (CREM): an essential factor for spermatogenesis in primates?. Int J Androl..

[r7] Ben-Jonathan N, Hnasko R (2001). Dopamine as a prolactin (PRL) inhibitor. Endocr Rev..

[r8] Blendy JA, Kaestner KH, Weinbauer GF, Nieschlag F, Schütz G (1996). Severe impairment of spermatogenesis in mice lacking the CREM gene. Nature.

[r9] Chong MY, Tan CH, Fujii S, Yang SY, Ungvari GS, Si T, Chung EK, Sim K, Tsang HY, Shinfuku N (2004). Antipsychotic drug prescription for schizophrenia in East Asia: rationale for change. Psychiatry Clin Neurosci..

[r10] Daniel PB, Rohrbach L, Habener JF (2000). Novel cyclic adenosine 3',5'-monophosphate (cAMP) response element modulator theta isoforms expressed by two newly identified cAMP-responsive promoters active in the testis. Endocrinology.

[r11] EMC - Electronic Medicines Compendium (2019). Chlorpromazine Hydrochloride 100mg/5ml Oral Syrup.

[r12] Fallah A, Mohammad-Hasani A, Colagar AH (2018). Zinc is an Essential Element for Male Fertility: A Review of Zn Roles in Men's Health, Germination, Sperm Quality, and Fertilization. J Reprod Infertil..

[r13] Freudenreich O (2012). Differential Diagnosis of Psychotic Symptoms: Medical Mimics.

[r14] Gellersen B, Kempf R, Sandhowe R, Weinbauer GF, Behr R (2002). Novel leader exons of the cyclic adenosine 3',5'-monophosphate response element modulator (CREM) gene, transcribed from promoters P3 and P4, are highly testis-specific in primates. Mol Hum Reprod..

[r15] He Z, Kokkinaki M, Pant D, Gallicano GI, Dym M (2009). Small RNA molecules in the regulation of spermatogenesis. Reproduction.

[r16] Healy D (2004). The Creation of Psychopharmacology.

[r17] Hogeveen KN, Sassone-Corsi P (2006). Regulation of gene expression in post-meiotic male germ cells: CREM-signalling pathways and male fertility. Hum Fertil (Camb).

[r18] Khazan N, Sulman FG, Winnik HZ (1960). Effect of reserpine on pituitary-gonadal axis. Proc Soc Exp Biol Med..

[r19] Kotaja N, Macho B, Sassone-Corsi P (2005). Microtubule-independent and protein kinase A-mediated function of kinesin KIF17b controls the intracellular transport of activator of CREM in testis (ACT). J Biol Chem..

[r20] Monaco L, Foulkes NS, Sassone-Corsi P (1995). Pituitary follicle-stimulating hormone (FSH) induces CREM gene expression in Sertoli cells: involvement in long-term desensitization of the FSH receptor. Proc Natl Acad Sci U S A..

[r21] Mosad SM, Abbas AS, Zalata AA (2000). Effect of reserpine (serpasil) on male fertility in rats. Mausoura J Forensic Med Clin Toxicol..

[r22] Nantel F, Monaco L, Foulkes NS, Masquilier D, LeMeur M, Henriksén K, Dierich A, Parvinen M, Sassone-Corsi P (1996). Spermiogenesis deficiency and germ-cell apoptosis in CREM-mutant mice. Nature.

[r23] Nur S, Adams CE (2016). Chlorpromazine versus reserpine for schizophrenia. Cochrane Database Syst Rev..

[r24] Ogunlesi M, Okiei W, Ofor E, Awonuga O (2009). Determination of the concentrations of zinc and vitamin C in oysters and some medicinal plants used to correct male factor infertility. J Nat Prod..

[r25] Oliva R (2006). Protamines and male infertility. Hum Reprod Update..

[r26] Orwa C, Mutua A, Kindt R, Jamnadass R, Anthony S (2009). Agroforestree.

[r27] Oyewopo A, Johnson O, Adeleke O, Oyewopo C (2018). Review on the role of glutathione on oxidative stress and infertility. JBRA Assist Reprod..

[r28] Raji Y, Ifabunmi SO, Akinsomisoye OS, Morakinyo AO, Oloyo AK (2005). Gonadal Responses to Antipsychotic Drugs: Chlorpromazine and Thioridazine Reversibly Suppress Testicular Functions in Albino Rats. Int J Pharmacol..

[r29] Sassone-Corsi P (1995). Transcription factors responsive to cAMP. Annu Rev Cell Dev Biol..

[r30] Sassone-Corsi P (1998). CREM: a master-switch governing male germ cells differentiation and apoptosis. Sem Cell Dev Biol..

[r31] Shabanian S, Farahbod F, Rafieian M, Ganji F, Adib A (2017). The effects of Vitamin C on sperm quality parameters in laboratory rats following long-term exposure to cyclophosphamide. J Adv Pharm Technol Res..

[r32] Sinclair S (2000). Male infertility: nutritional and environmental considerations. Altern Med Rev..

[r33] Steger K (1999). Transcriptional and translational regulation of gene expressions in haploid spermatids. Anat Embryol (Berl).

[r34] Steger K, Failing K, Klonisch T, Behre HM, Manning M, Weidner W, Hertle L, Bergmann M, Kliesch S (2001). Round spermatids from infertile men exhibit decreased protamine-1 and -2 mRNA. Hum Reprod..

[r35] Weinbauer GF, Behr R, Bergman M, Nieschlag E (1998). Testicular cAMP responsive element modulator (CREM) protein is expressed in round spermatids but is absent or reduced in men with round spermatid maturation arrest. Mol Hum Reprod..

[r36] Weiner N, Molinoff PB, Siegel G, Agranoff B, Albers RW, Molinoff P (1989). Catecholamines. Basic Neurochemistry.

[r37] WHO - World Health Organization (2003). Essential Medicines.

[r38] Zamani Z, Zare S, Sadrkhanlou R, Ahmadi A, Movahed E (2015). The effects of chlorpromazine on reproductive system and function in female rats. Int J Fertil Steril..

